# State Medical Society

**Published:** 1857-06

**Authors:** 


### State Medical Society.

We hope the annual meeting of our State Society will not be forgotten, for we would fain see a large attendance. Let us urge upon the committees the importance of being prepared with their reports, and let all go prepared to take part in the proceedings, in order that the meeting may be made as interesting and profitable as possible. Iowa has a large number of medical men, and among them many who are as capable as those of any of our sister states, to make her State Society meetings occasions of interest, and its results as valuable to the advance of medical knowledge. Let them only put their shoulders to the wheel and its "transactions" will rank, as they should, equal in worth to those of other State Societies. The meeting will be held at Iowa City, commencing on Wednesday, June 10th, 1857.

